# DNA damage checkpoint adaptation genes are required for division of cells harbouring eroded telomeres

**DOI:** 10.15698/mic2015.10.229

**Published:** 2015-09-21

**Authors:** Sofiane Y. Mersaoui, Serge Gravel, Victor Karpov, Raymund J. Wellinger

**Affiliations:** 1 Dept of Microbiology and Infectious Diseases, Faculty of Medicine and Health Sciences, Université de Sherbrooke, 3201, Rue Jean Mignault, Sherbrooke, J1E 4K8, Canada.

**Keywords:** telomeres, DNA damage checkpoints, chromosome capping

## Abstract

In budding yeast, telomerase and the Cdc13p protein are two key players acting to ensure telomere stability. In the absence of telomerase, cells eventually enter a growth arrest which only few can overcome via a conserved process; such cells are called survivors. Survivors rely on homologous recombination-dependent mechanisms for telomeric repeat addition. Previously, we showed that such survivor cells also manage to bypass the loss of the essential Cdc13p protein to give rise to Cdc13-independent (or cap-independent) strains. Here we show that Cdc13-independent cells grow with persistently recognized DNA damage, which does not however result in a checkpoint activation; thus no defect in cell cycle progression is detectable. The absence of checkpoint signalling rather is due to the accumulation of mutations in checkpoint genes such as *RAD24* or *MEC1*. Importantly, our results also show that cells that have lost the ability to adapt to persistent DNA damage, also are very much impaired in generating cap-independent cells. Altogether, these results show that while the capping process can be flexible, it takes a very specific genetic setup to allow a change from canonical capping to alternative capping. We hypothesize that in the alternative capping mode, genome integrity mechanisms are abrogated, which could cause increased mutation frequencies. These results from yeast have clear parallels in transformed human cancer cells and offer deeper insights into processes operating in pre-cancerous human cells that harbour eroded telomeres.

## INTRODUCTION

Telomeres are essential for genome stability in all organisms with linear chromosomes; they play multiple roles in chromosome end protection, chromosome end replication and distinguishing chromosome ends from double strand breaks (DSBs) 1,2. Indeed, chromosome ends and DSBs superficially share a great deal of similarity as both are physical ends of DNA molecules. However, functional telomeres do not activate checkpoints and they are not subjected to DNA repair activities such as homologous recombination (HR) or end-to-end fusions 3,4. These features are provided by the unique structures and organization of the nucleoprotein complexes located at the ends of chromosomes 5,6. Telomere structure and the molecular functions required for the above activities are also highly conserved, suggesting a common evolutionary origin. Telomeric DNA consists of short tandem DNA repeats, which generally create a G-rich strand that makes up the 3’ end of the chromosome. This strand also protrudes beyond the 5’ end, forming a single stranded “G-tail” 7,8,9.

Chromosomes of *Saccharomyces cerevisiae *cells share these features and serve as an excellent model for studying telomere biology. There is an approximately 300 bps double-stranded portion and a short single-stranded DNA portion of characteristic repeats at each chromosome end. These repeats are associated with specialized proteins that are essential for telomeric functions 1. Specifically, telomeric capping in yeast is assured by a heterotrimeric complex composed of Cdc13p, Stn1p and Ten1p (the CST-complex; 1,10,11,12,13). Of these, Cdc13p specifically recognizes a short telomeric G-rich DNA substrate that can be the terminal G-tail and all three genes are essential. Hypomorphic alleles of *CDC13 *exist and the *cdc13-1 *allele, for example, confers temperature sensitivity (ts) to cells 14. In cells with the *cdc13-1 *allele that are incubated at restrictive temperatures (>26°C), the C-rich strand of telomeric DNA is degraded, yielding extensive single-stranded DNA that can reach into subtelomeric DNA 15. These ends become recognized as sites of DNA damage which triggers a robust Rad9-dependent cell-cycle checkpoint response and, eventually, cell death 13. Cdc13p is also involved in allowing the complete replication of telomeres by recruitment of telomerase to telomeres 16.

Telomerase is the ribonucleoprotein that elongates telomeres during S-phase to counteract the shortening of telomeric DNA that occurs due to the ‘end replication problem’ 17,18,19. A loss of telomerase leads to progressive telomere shortening, the so-called “ever shorter telomeres”, or “EST” phenotype and a concomitant loss of the telomeric capping function 20,21,22. Upon outgrowth of such cultures, the majority of cells ceases to divide after approximately 60-80 generations.

However, a small proportion of cells can regain the ability to divide and to maintain chromosome ends by telomerase-independent mechanisms; these cells are called survivors 1,23. There are two major types of survivors which have one of two different arrangements of telomeric and subtelomeric DNA 24. In type I survivors, a complex subtelomeric repeat element called Y’ spreads to all telomeres and only a very short telomeric repeat tract remains. Type II survivors on the other hand are defined by the presence of very long and heterogeneous telomeric repeat tracts 24. These two types of cells can also be distinguished by the genetic requirements for the pathways involved 25. Survivor cells still require Cdc13p-mediated chromosome capping, but Cdc13-independent survivors can be generated 26,27,28. These latter cells do grow without the canonical organization of chromosomal ends; their telomeres yield a new pattern of terminal restriction fragments (TRFs) with a complete absence of discrete bands. Moreover, there is a high amount of telomeric and subtelomeric single-stranded DNA at chromosomal ends in Cdc13-independent survivors 28. Previous evidence also suggests that telomeres in these cells essentially are elongated and maintained by HR mechanisms 28,29. Finally, cell growth without the capping protein Cdc13p is possible if genes involved in DSB processing (*EXO1*, *SGS1*) and certain checkpoint genes (*RAD9*, *RAD24*) are deleted simultaneously 29.

It has been speculated that particularly type II survivor yeast cells resemble human cancerous cells that replenish their telomeric repeat DNA via alternative telomerase-independent mechanisms (alternative lengthening of telomeres, or ALT), 30,31. A commonality between yeast survivor and human ALT cells is that both are thought to amplify their telomeric DNA through recombination-dependent DNA replication 32. This probably occurs through a mechanism involving extrachromosomal circular DNA containing telomeric repeat sequences 28,33. However, in virtually all human transformed cells, genomic integrity mechanisms are severely hampered and cells divide with ongoing genomic instability 34.

Here we show that Cdc13-independent survivor cells (cap-independent cells) do grow even though DNA damage foci can be observed to persist on telomeres. However, this apparent damage does not result in checkpoint signalling or cell cycle arrest. Our results show that this is because the Mec1-branch of the damage signalling pathway was abrogated via mutations in central checkpoint genes, mainly *RAD24* or *MEC1*. We also report that *CDC5*, *PTC2* and *TID1* are required for the initial generation of cap-independent cells. Mutations in either of these genes significantly reduce the ability of survivor cells to overcome the loss of Cdc13p and resume growth. These results therefore reveal intriguing similarities between yeast cells dividing in the absence of Cdc13p and human cancerous ALT-cells. Both display absence of cell division controls and continued cell divisions, despite ongoing telomere instability. We therefore hypothesize that this yeast system represents a useful tool for investigating the early phases of human cancerous cell growth.

## RESULTS

### Permanent detection of telomeric DNA damage but no checkpoint activation in Cdc13-independent survivors

**Figure 1:**
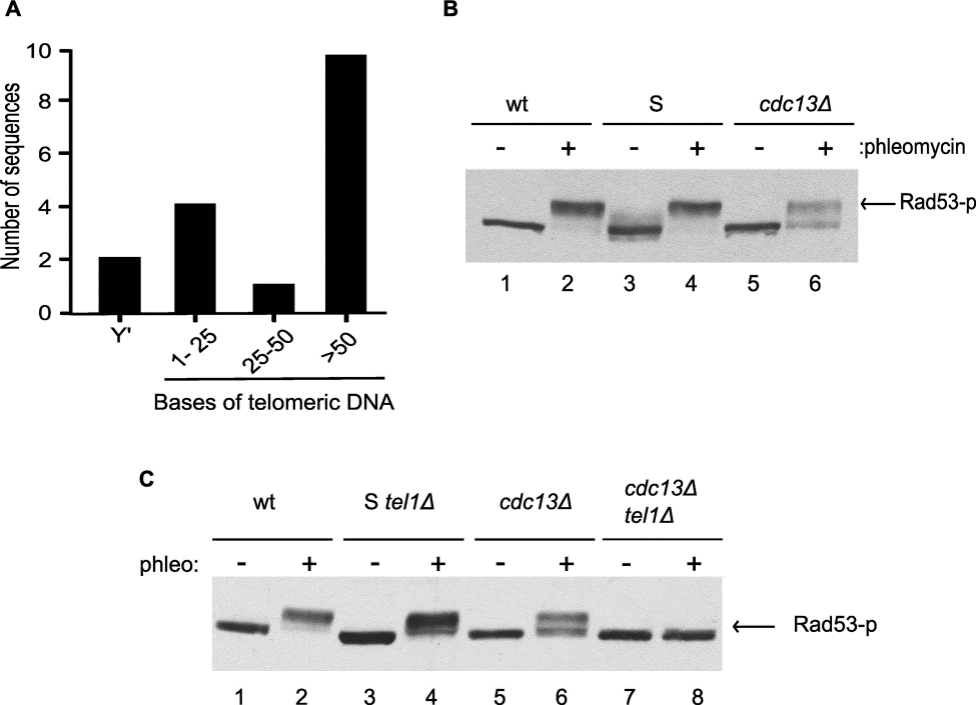
FIGURE 1: Mec1-dependent Rad53 phosphorylation is defective in *cdc13*Δ cells. **(A)** Terminal chromosomal regions were amplified by PCR and sequenced. Telomeric DNA (in bases) present on the amplified fragments is indicated. A total of 17 independent sequences were obtained, with two of them ending in the subtelomeric Y’ element. **(B)** Exponentially growing wild-type (wt), Survivor (S) or *cdc13*Δ (*cdc13*Δ) cells were left untreated or were treated with phleomycin for 2 h. Protein extracts were prepared and analyzed by western blotting with an anti-Rad53 antibody. **(C)** As in (B) except that cells also lacked the *TEL1 *gene as indicated in the Survivors (S) and Cdc13-independent (*cdc13*Δ) cells. Cells were left untreated or were treated with phleomycin for 2 h.

Previous analyses of telomeres in Cdc13-independent survivors showed that their TRFs are extremely heterogeneous in length (see Fig. S1, lanes 8-10; and 28). In order to obtain a more precise assessment of the terminal sequences on their chromosomes, we cloned and sequenced 17 independent terminal DNA fragments. 10 of those 17 harboured potentially functional telomeric repeat tracts (> 50 bps of repeat DNA), one had a critically short tract (35 bps) and six had tracts that were too short for even a single binding site for Rap1p, the major yeast protein binding double-stranded telomeric repeat tracts 1. In fact, two of the 17 clones had no detectable telomeric G-rich sequences and ended with a subtelomeric Y’ element (Fig. 1A).

**Figure 2:**
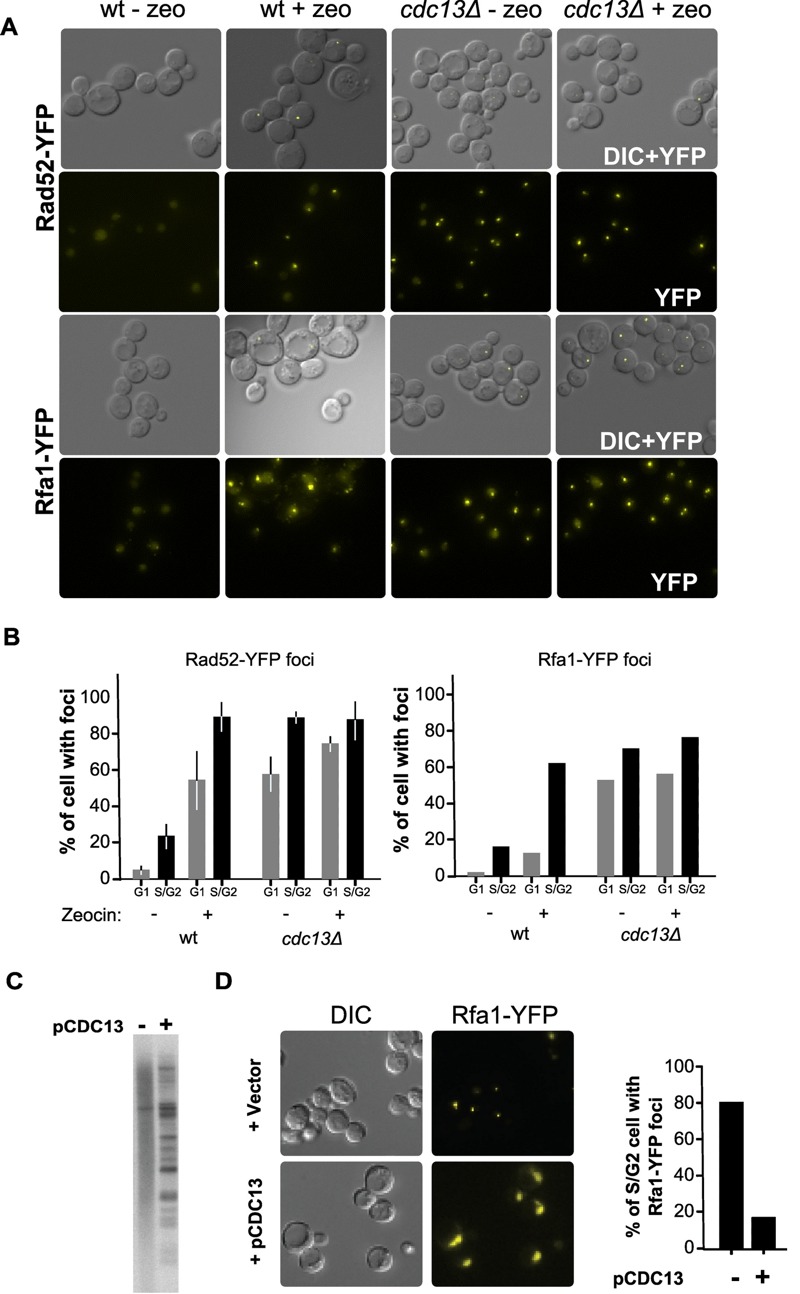
FIGURE 2: Rad52p and Rfa1p proteins form DNA damage foci in untreated *cdc13*Δ cells. **(A)** Wild-type (wt) or *cdc13*Δ cells expressing the Rfa1-YFP fusion protein (RWY801) or the Rad52-YFP protein (CPY821) were analysed by fluorescence microscopy untreated or treated with zeocin for 3 h. Representative examples of the images obtained are shown. **(B) **Quantification of cells with foci. Cells were morphologically divided into G1 cells (unbudded cells) and G2M cells (large budded cells). The Rad52-YFP experiment was performed in 3 independent biological replicates, and the Rfa1-YFP experiment was performed with at least 150 cells for each condition. **(C)** Southern blot with DNA derived from MLY122 using a telomeric DNA probe. Cells were growing in the absence of Cdc13p (lane marked -) or the wt *CDC13* gene was introduced via plasmid p*CDC13 *(lane marked +). Note that re-introduction of the protein CDC13 restores survivor type II TRF phenotype. **(D)** The re-expression of *CDC13* eliminates the formation of Rfa1-YFP foci in *cdc13*Δ cells. Strain CPY821 was grown to become Cdc13-independent and then an empty vector or pCDC13 was introduced. Representative examples of images obtained as in (A) are shown in the left panel. Quantification of the data is shown in the right panel. >100 cells were counted for each sample.

Cells with such short or absent telomeric repeat tracts and potentially exposed single-stranded Y’-DNA at chromosome ends display a strong DNA damage checkpoint 35. We therefore verified whether the DNA damage checkpoint was activated in Cdc13-independent survivor cells, by assessing the level of phosphorylation of Rad53p, a major DNA damage checkpoint effector kinase in budding yeast (36; Fig. 1B). Rad53p migrates as a single discreet band in unperturbed growing cells, while DSBs caused by the addition of the radiomimetic phleomycin (a derivative of bleomycin) causes DNA damage checkpoint activation and robust phosphorylation of Rad53p as detected in the form of a retarded Rad53p band (Fig. 1B, lane 2). In Cdc13-independent survivors, no phosphorylated Rad53p is detected in unperturbed cells and phleomycin addition only causes a very partial retardation of Rad53p, as if only a partial phosphorylation was possible (Fig. 2B, lanes 5, 6).

Similar results were obtained when phosphorylation of the checkpoint effector kinase Chk1p was assessed (Fig. S2). DNA damage, once detected, causes the activation of two sensor kinases, namely Mec1p and Tel1p (ATR and ATM in mammals 37,38), and phleomycin induced damage is sensed mainly via Mec1p. We thus reasoned that the very partial Rad53p phosphorylation detected in Cdc13-independent survivors could be due to a loss of the Mec1 branch of the sensor kinase pathway. We therefore constructed Cdc13-independent survivor strains that lacked Tel1p and assessed the degree of Rad53p phosphorylation induced by phleomycin. While survivor strains with a *tel1*Δ allele are able to strongly phosphorylate Rad53p (Fig. 1C, lane 4), Cdc13-independent survivors harbouring the *tel1*Δ allele were unable to do so (Fig. 1C, lane 8).

As DSBs are usually recognized rather efficiently, we wondered whether telomeres in Cdc13-independent cells had developed an alternative, telomeric DNA-independent capping mechanism or whether they were simply ignored by the DNA damage/repair machinery. To address this, we used fluorescence microscopy to visualize proteins involved in the recognition and repair of DNA DSBs. DNA end resection at DSB generates single-stranded DNA (ssDNA) that is rapidly bound by the RPA complex, and the focal localization of RPA on DNA damage sites can be visualized in living cells via YFP-tagged Rfa1p 39. As a positive control, we treated cells with the radiomimetic drug zeocin and, as expected, zeocin induced the formation of Rfa1-YFP foci in wild-type cells (Fig. 2A, B). Strikingly, in Cdc13-independent survivor cells, Rfa1-YFP foci were readily detectable, even in the absence of zeocin treatment (Fig. 2).

Quantification indicates that ssDNA is present in more than 80% of unperturbed S/G2 cells and more than 50% of cells in G1. As an additional read-out for the presence of DNA damage recognition, we carried-out the same analysis with Rad52-YFP. In untreated wt (wild type) cells, Rad52-YFP foci are rare and zeocin treatment increased the fraction of cells displaying Rad52-YFP foci to about 80-90 % (Fig. 2). Again in stark contrast with these results, the majority of untreated Cdc13-independent cells already had Rad52-YFP foci and there was no detectable increase upon zeocin treatment.

If uncapped telomeres were the source of Rfa1-YFP foci in Cdc13-independent survivor cells, they should rapidly disappear following reintroduction of *CDC13*. We thus transformed Cdc13-independent survivor cells with a plasmid that contained the wt *CDC13 *gene and assessed telomeric restriction fragments and RPA-foci. TRF patterns in Cdc13-independent survivor cells with Cdc13p re-expressed reverted to a typical survivor pattern (compare Fig. 2C with Fig. S1) and the appearance of Rfa1-YFP foci reverted back to a level observed in *CDC13* wt cells (Fig. 2D). These results indicate that re-establishing a Cdc13-dependent capping system in *cdc13*Δ cells eliminates the presence of the detected DNA damage and suggest that telomere uncapping is indeed the source of the RFA and Rad52p foci in *cdc13*Δ mutants. Thus, in Cdc13-independent survivors, uncapped and resected telomeres persist throughout the cell cycle and are bound by proteins that would allow ongoing DNA repair. However, Mec1-mediated DNA damage signalling is by and large abrogated, allowing for cell divisions to continue and hence, culture growth.

### A functional checkpoint is incompatible with growth in the absence of CDC13

The data described above indicate that uncapped telomeres and apparent DNA damage foci are constitutively present in a large fraction of the Cdc13-independent cells. Given the virtual absence of DNA damage signalling, we considered that inactivation of Mec1-mediated checkpoint signalling could have been caused by reduced or dysregulated DNA resection. To test this, an HO endonuclease-induced DSB was created in cells in such a way that no homologous sequences for recombinational repair were present next to the break. The rate of DNA resection next to the HO site was monitored by denaturing slot blot analysis using a probe complementary to the processed DNA strand 40.

In this assay, signal loss for the resected strand was very similar in *CDC13 *cells and cells that harboured a *cdc13*Δ allele (Fig. S3A, S3B). As an additional assay for DNA resection and ensuing repair, we assessed completion of mating-type switching (a process based on HR) by Southern blot analyses. As observed in the resection assay, no significant difference between wt and cells that lack Cdc13p could be detected (Fig. S3C). These physical assays show that the dynamics and efficiency of DNA end-processing and HR repair remained virtually unaffected in cells growing without Cdc13p.

**Figure 3:**
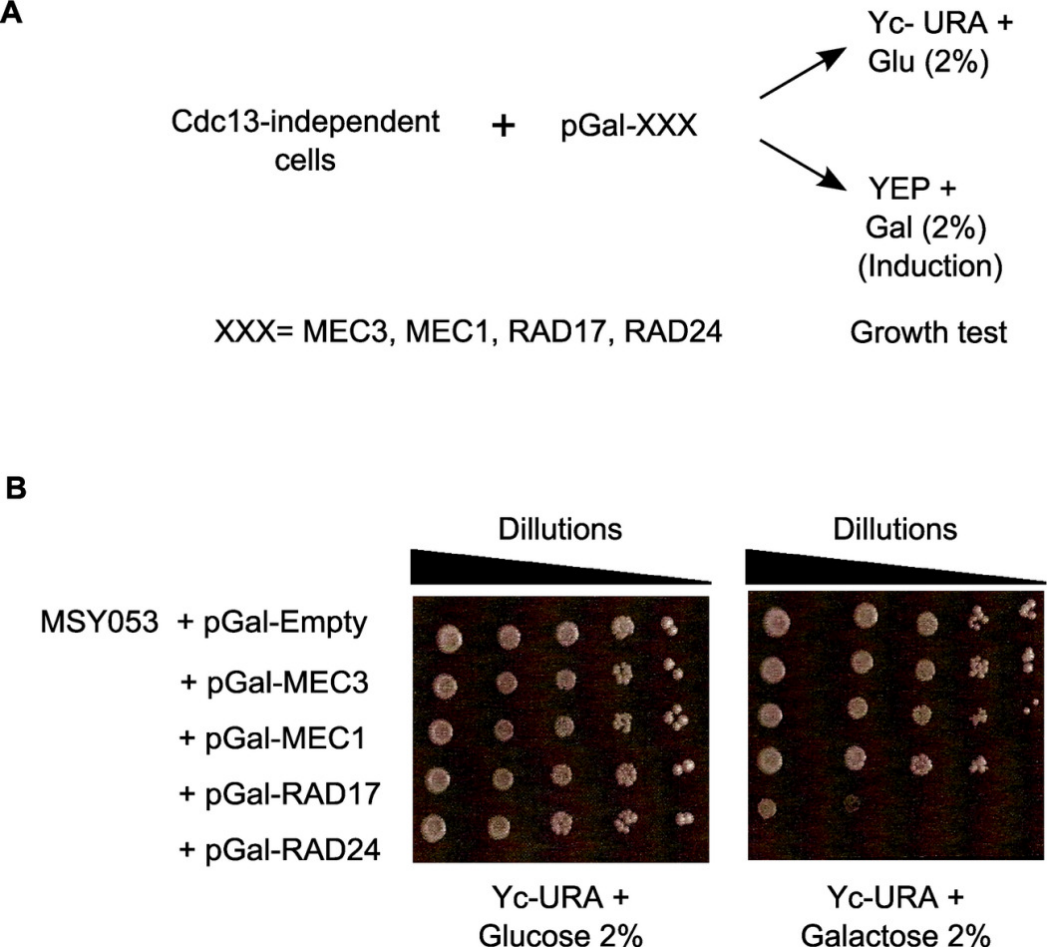
FIGURE 3: An abrogated Mec1-branch of the checkpoint pathway is required for Cdc13-independent cell growth. **(A)** Schematic representation of the functional complementation test: 12 independent strains of *cdc13*Δ cells were used in this experiment. Each cap-independent *cdc13*Δ strain was transformed with five constructs (plasmids): pGal-*MEC1*, pGal-*MEC3*, pGal-*RAD17*, pGal-*RAD24* or empty vector pGal-Empty as a control. Serial dilutions of cultures of the resulting strains were spotted onto YEP+ Glucose (2%) for growth control and onto YEP+ Galactose (2%) to induce the expression of indicated genes. **(B)** Cells of a Cdc13-independent strain stop growing when the mutated checkpoint gene is complemented by the corresponding wild-type construct. The results shown in this figure were derived with strain MSY053 which holds a mutation in *RAD24 *in the genome.

An alternative hypothesis to explain the absence of Mec1p-mediated DNA damage signalling is that the checkpoint signalling proteins are themselves impaired, for instance by having acquired a mutation. In this case a forced re-expression of the functional wt allele of a mutated gene would cause a growth arrest in cells with perceived permanent DNA damage. We therefore cloned the wt alleles of genes encoding essential components of the Mec1-checkpoint pathway into a vector that allowed a carbon source dependent expression of the protein (pGal; Fig. 3A). Twelve individual and independently generated Cdc13-independent survivor strains were then transformed with those plasmids and the resulting growth behaviour was assessed on plates that induced expression of the particular gene (Gal-plates).

Remarkably, each of the twelve strains showed a growth arrest with one of the plasmids used. An example is shown in Fig. 3B, where strain MSY053 grew well on Gal-plates when *MEC1,* *RAD17 *or *MEC3 *were overexpressed, yet they failed to grow when *RAD24 *was overexpressed (Fig. 3B, right). These cells did grow on glucose plates (Fig. 3B, left) and Rad24p overexpression was readily tolerated in wt cells or regular survivor cells that contained a wt copy of *CDC13* (Fig. S4, top plate). The genomic *RAD24 *locus of strain MSY053 was sequenced and found to contain a frameshift mutation that caused a loss of function. In summary, in the 12 independently obtained Cdc13-independent survivor strains, sensitivity to Rad24p expression was uncovered seven times, Mec1p six times, Rad17p two times and to Mec3p one time. These results thus further confirm that persistent telomere uncapping is sensed as DNA damage in cells without the Cdc13 protein and that in the absence of Cdc13p, cells can only grow with an inactivated Mec1-branch of checkpoint signalling.

### Factors required for adaptation to DSBs are required for growth in the absence of Cdc13p

**Figure 4:**
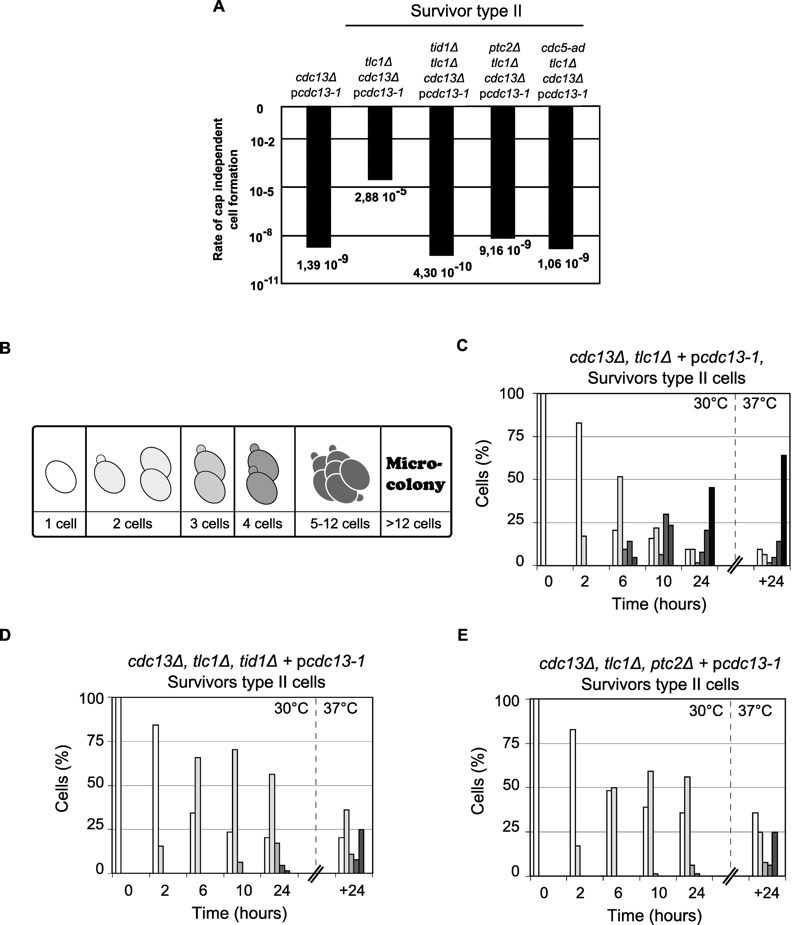
FIGURE 4: The formation of *cdc13*-independent survivors (*cdc13*Δ) requires DSB adaptation genes. **(A)** The rate of successful colony formation was calculated by fluctuation test foAr five strains: (i) telomerase positive controls, *cdc13*Δ + pcdc13-1 (VKY20), (ii) type II survivors: *tlc1*Δ*, cdc13*Δ + pcdc13-1 (MLY112), (iii) type II survivors harbouring a deletion of *TID1:* *tid1*Δ*, tlc1*Δ*, cdc13*Δ + pcdc13-1 (VKY19), (iv) type II survivors harbouring a deletion of *PTC2: ptc2*Δ*, tlc1*Δ*, cdc13*Δ + pcdc13-1 (VKY12), or (v) type II survivors harbouring the *cdc5-ad* allele: *cdc5-ad, tlc1*Δ*, cdc13*Δ + pcdc13-1 (MSY421). **(B)** Schematic representation of standardization for morphological classification used to analyse cell cycle progression of the strains of interest. **(C) (D) (E)** Single round unbudded cells (64 cells) from three strains described above (ii) (iii) (iv) were identified and arrayed on YEPD plates and incubated at 30°C (restrictive temperature for the *cdc13-1 *allele). Morphology and growth of cells were inspected microscopically at 2, 6, 10 and 24 h after incubation. Plates were then incubated at 37°C for another 24 h. Colony morphology was recorded as outlined in (B) and results plotted as the percentage of the initial group. **(C) **Cell cycle progression for the survivor type II *tlc1*Δ*, cdc13*Δ + pcdc13-1 (MLY112); **(D) **and** (E)** cell cycle progression for both adaptation deficient mutants *tid1*Δ*, tlc1*Δ*, cdc13*Δ + pcdc13-1 (VKY19), *ptc2*Δ*, tlc1*Δ*, cdc13*Δ + pcdc13-1 (VKY12).

It has been observed that wt cells are only very rarely able to overcome a complete loss of Cdc13p but actual survival rates are unknown. We used fluctuation analyses to measure survival rates of wt cells and of cells that have overcome a telomerase deficiency (survivor cells). Only about 1 in 10^9 ^- 10^10^ cells survived an abrupt loss of Cdc13p and created a growing culture in telomerase-positive cells, whereas in cultures of survivor cells the rate is at least 1000 fold higher (Fig. 4A, 28). This difference may be explained by the fact that telomerase-mediated telomere maintenance is not required in survivors and that upon loss of Cdc13p, cells only have to adapt to chromosome capping loss. Loss of telomeric capping is thought to have comparable effects as induction of a number of DSBs at the same time, but direct data on this is lacking.

As a consequence of telomere loss, mammalian and budding yeast cells arrest the cell cycle for prolonged periods of time before resuming growth, even in the presence of persistent damage. The latter process has been dubbed ‘adaptation’ and *CDC5, TID1* and *PTC2* genes are key elements required for adaptation to occur in yeast 37. We therefore examined whether the generation of Cdc13-independent survivors is dependent on adaptation genes. Fluctuation analyses show that survivor cells that also harbour a deletion of *TID1* or *PTC2 *(*cdc13*Δ* tcl1*Δ* tid1*Δ cells or *cdc13*Δ* tcl1*Δ* ptc2*Δ cells), as well as survivors that harbour an adaptation negative *CDC5 *allele (*cdc13*Δ* tcl1*Δ* cdc5-ad *cells) only generate survivors extremely rarely, at a rate of about 5 x 10^-9^ to 5 x 10^-10 ^(Fig. 4A). Similarly, when the morphology of cells and the generation of micro-colonies was examined in a single cell assay after inactivation of Cdc13p via a temperature upshift to restrictive temperatures, survivor cells (*cdc13*Δ* tcl1*Δ + pcdc13-1) showed clear signs of adaptation; after 24 h at 30°C, more than 50% of the cells had grown into micro-colonies of 5 cells or more and at least 60% of them became micro-colonies with more than 12 cells after another 24 h at 37°C (Fig. 4B, C). In contrast, more than 50% of adaptation-negative survivors (*cdc13*Δ* tcl1*Δ* tid1*Δ + pcdc13-1 cells or *cdc13*Δ* tcl1*Δ* ptc2*Δ + pcdc13-1 cells) were still single cells or small budded cells after 24 h at 30°C and only a small minority formed 5-12 cell colonies (Fig. 4D, E). Finally, none of the 64 adaptation-negative (either *ptc2*Δ or* tid1*Δ) survivor cells generated a viable colony. This was expected given the very low rate determined by fluctuation tests. We conclude that the *TID1*, *PTC2 *and *CDC5 *genes are required in yeast for both, adaptation to persistent DNA damage after DSB induction as well as for the ability of survivor cells to generate growing colonies after the loss of telomere capping.

### A reversible component to adaptation to telomere uncapping

**Figure 5:**
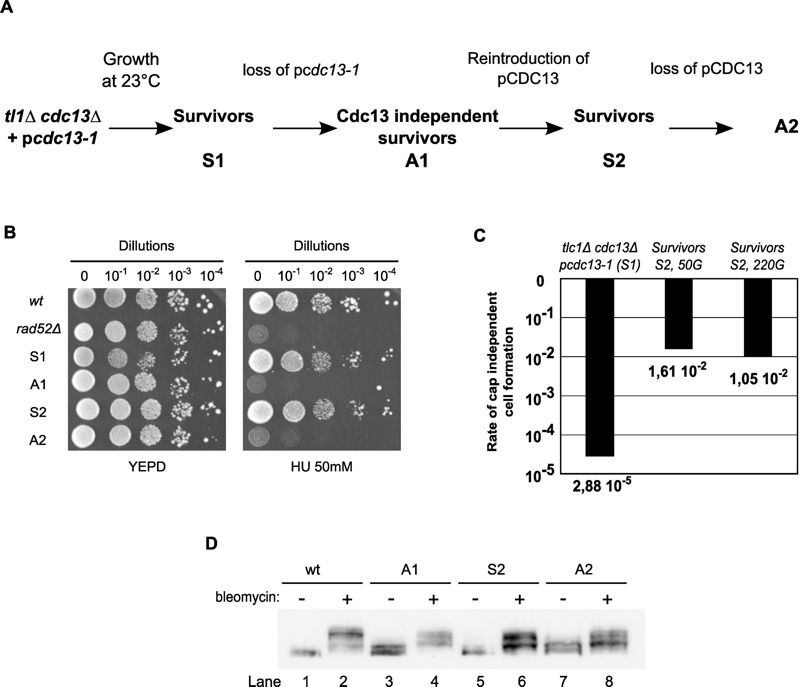
FIGURE 5: The process of generating *Cdc13*-independent survivors (*cdc13*Δ-cells) comprises a reversible component. **(A)** Schematic representation of the procedure used to generate S2 survivors and second generation *cdc13*Δ-A2 of Cdc13-independent survivors. S1 survivors and *cdc13*Δ-A1 were generated as described in Fig. 1. S2 survivor cells were derived from *cdc13*Δ-A1. These cells were grown for up to 220 generations (220G) after transformation with the plasmid carrying the *cdc13-1* allele. For *cdc13*Δ-A2 cells (MLY123), the loss of the p*CDC13 *plasmid was verified by growing cells on FOA synthetic medium. **(B) **Cells identified with symbols as in (A) were spotted onto YEPD plates with or without hydroxyurea (HU) and incubated for 72 h at 23°C. A haploid *rad52*Δ strain was used as control for HU sensitivity. **(C) **Rate of successful *cdc13*-independent cell formation (calculated by fluctuation test) for survivors S1 (MLY112) and survivors S2 (MLY122 + pcdc13-1). S2 survivors were grown for 50 or 220 generations after p*cdc13-1* introduction and before the fluctuation test for plasmid loss was performed. **(D)** Western blot of whole cell protein extracts prepared from strains indicated with the same symbols as in (A)(top). Cell treatment with bleomycin is indicated with - and +, and the blot was probed with an anti-Rad52 antibody as in Fig. 1.

The results thus suggest that adaptation to telomere uncapping requires known adaptation genes, that telomere maintenance is carried out by recombination, and that at least one gene of the DNA checkpoint signalling machinery has to be inactivated by mutation. However, it was unclear whether this adapted state, once achieved as a metabolic state, is stable and remains active in cells or whether it is reversible. In order to assess this question, we generated cells that passed through the survivor state and had adapted to Cdc13p-loss. We then re-introduced the Cdc13p capping protein into these cells to let them grow with the capping protein (see schematic in Fig. 5A). The TRF patterns of such cells changed and again became typical for survivor cells 28.

Adapted *cdc13*Δ cells were sensitive to agents causing replication stress, such as hydroxyurea (HU). However after Cdc13p re-introduction they reverted to an insensitive phenotype similar to wt and pre-adaptation survivors (Fig. 5B, 28). We eventually challenged these latter cells to a Cdc13p-loss a second time. In this experiment, we call survivors before the first Cdc13-loss ‘S1-cells’ and those generated by re-introduction of Cdc13p into Cdc13-independent cells ‘S2-cells’ (see Fig. 5A). We then compared the rates of adaptation of S1 or S2 cells by fluctuation analyses (Fig. 5C). Remarkably, even though S2 cells had been derived from adapted cells, only about 1 % of them formed viable colonies after the second loss of Cdc13p. While this rate was about 1000 fold higher than that observed for the original transition of S1 cells to adapted cells (Fig. 4A, 5C), it still far from complete and indicated that the reintroduction of Cdc13p into adapted cells is associated with a reversion of at least part of the phenotype.

This low adaptation rate did not change with longer outgrowth of S2 cells; after 220 generations of growth in the S2 state, the rate of adaptation remained about 1 % (Fig. 5C). Similar to the transition from S1 to the first adaptation state, the transition from S2 to adapted state also resulted in an increased sensitivity to hydroxyurea (HU) (Fig. 5B). However, the capacity to fully phosphorylate Rad53p did not recover after reintroduction of Cdc13p into adapted A1 cells (S2 cells; Fig. 5D, lane 6). The signalling also remained partial after the second loss of Cdc13p (Fig. 5D, lane 8), as would be expected if this part of the phenotype was genetically determined. We conclude that the very low rate of colony formation after a first loss of Cdc13p from survivor cells (about 3x10^-5^) can be explained by the fact that a mutation in one of the Mec1-branch checkpoint genes is required. A second Cdc13p loss after a temporary reintroduction of it still is poorly tolerated and only about one percent of cells survive, suggesting that a metabolic, non-genetic, component of the adaptation state does reverse upon introduction of Cdc13p.

## DISCUSSION

Checkpoint adaptation was originally described as the ability of *S. cerevisiae* cells to overcome a sustained checkpoint arrest due the presence of irreparable DNA damage 41,42,43. Subsequently, mechanisms to abrogate a prolonged checkpoint arrest were also reported to operate in *Xenopus laevis* and human cells and the well conserved genetic requirements for the process suggested a common evolutionary origin (44,45; see below). Although cells undergoing checkpoint adaptation almost invariantly die in subsequent cell cycles, owing to rampant genome instability, some cells do divide a limited number of times. However, it remained unclear how these cells managed to pass through the cell divisions and whether checkpoint abrogation is permanent or temporary. A conceptually similar situation arises in the etiology of malignant human cells. Current evidence strongly suggests that precancerous cells, very early on, undergo a phase of high level genome instability that is due to dysfunctional telomeres 46. Once this serious bottleneck is overcome, cancerous cells have invariably activated a mechanism to maintain telomeric repeats, which is almost always achieved by a reactivation of telomerase, and they have inactivated genome surveillance mechanisms, in most cases at least including TP53 34. Experimental setups that allow a systematic study of the chain of events happening in human cells when passing from normal to pre-cancerous therefore promise to yield invaluable insights into the very early etiology of cellular transformation.

Budding yeast cells maintain telomeres via a constitutively active telomerase, but cells can be engineered to lose telomerase and thus, in this respect, phenotypically become more like human somatic cells 1. For example, yeast cells without telomerase endure telomere shortening eventually leading to crisis and growth arrest, when at least some telomeres are dysfunctional 1. Yeast survivor cells are defined as the fraction of telomerase negative yeast cells that overcome this short telomere crisis by replenishing telomeric repeats by HR. Previously, we showed that again only a fraction of such survivor cells are able to survive a loss of functional telomere capping as well, generating so called cap-independent survivors 28. These latter cells are able to divide but are very sensitive to genotoxic compounds and they have a significantly reduced ability for DNA damage signalling.

The characteristics of the cap-independent survivor cells reported here are similar in several ways to early transformed human cells. Most significantly, cap-independent survivors have acquired mutations in at least one gene in the canonical DSB signalling pathway, which is governed by Mec1p in yeast (ATR in humans; Fig. 3, S4). As a consequence, the downstream effectors for cell cycle arrest are not activated and cell divisions continue even in the continued presence of DNA damage. The fact that cells stopped growing when we re-established the pathway by transforming cells with wt copies of the mutated genes demonstrates that these continued cell divisions are indeed dependent on an abrogated damage signalling pathway (Fig. 3). A conceptually similar effect has been reported in human and mouse cancer cells in which the reversion of a mutated TP53 allele leads to a re-establishment of genome surveillance mechanisms and tumour cell death 47,48,49. As in human cancer cells, the physical recognition of a DSB and its actual repair appear virtually unaffected in the cap-independent yeast cells (Figs. S3). Remarkably, a very high level of DNA damage foci are observed in cap-independent survivor cells in any phase of the cell cycle (Fig. 2). In normal cells, this level of DNA damage would invariably lead to a prolonged cell cycle arrest in G2/M 39. However, this is not the case in Cdc13p-independent cells due to the loss of damage signalling.

The results strongly suggest that the DNA damage foci detected in these experiments are on chromosome ends and that DNA repair activity, i.e. HR, is ongoing on those sites. This idea is supported by the observations that a significant fraction of telomeric restriction fragments cloned from these cells does not contain any telomeric sequences anymore (Fig. 1). Those sites are therefore indistinguishable from any other DSB in the genome and are presumably recognized as such. Furthermore, when we introduced one additional specific DSB via an induced endonucleolytic cleavage, that DSB gets resected and eventually repaired via HR with similar kinetics to normal cells (Fig. S3). Thus, DNA damage in these cells is recognized by the DNA repair machinery and HR particularly, can be active.

The reason why Cdc13p-independent cells remain viable and the cultures grow with abundant DNA damage foci present is that in these cells, telomeric DNA (either subtelomeric complex repeats or terminal short repeats) becomes amplified and chromosome ends eventually are all composed of extensive head to tail arrays of repeated elements 1. Therefore, the genome remains, by and large, intact and functional, even though significant amounts of terminal DNA may be lost due to end-degradation from these repeated elements.

A similar dynamic behaviour of DNA adjacent to telomeres has also been described in *Drosophila melanogaster* 50. In this organism, chromosomes end with telomere specific retroelements, i.e. arrays of repeated and complex DNA elements, and there are neither short direct repeats nor evidence for a telomerase enzyme. As expected therefore, the terminal elements suffer gradual sequence losses with each cell division. However, the relatively limited overall losses are confined to the most distal parts of the chromosomes and these are balanced by the occasional acquisition of large repeat units 50. Despite this very dynamic telomere maintenance mechanism, the genome in *D. melanogaster* cells remains intact, as it does in the cap-independent yeast cells reported here.

During these studies of cap-independent yeast cells, we further discovered that genes required for checkpoint adaptation, namely *CDC5wt, PTC2 *and *TID1/RDH54*, are also required for generating dividing survivor cells that harbour a non-functional *cdc13 *allele (Fig. 4). Ptc2p is a type 2C phosphatase and has been described as being directly involved in deactivating the DNA damage signalling pathway by dephosphorylating proteins, in particular Rad53p 51. The cellular regulation of this phosphatase and how its checkpoint-abrogating activity is induced remain unclear however. The Tid1/Rdh54p protein is involved in DSB repair, by mitotic and meiotic HR, and it is itself phosphorylated by the Mec1p branch of the checkpoint signalling pathway after DNA damage 52. The precise roles for Tid1/Rdh54p in checkpoint adaptation are poorly defined though.

Consistent with the above, the adaptation characteristic *cdc5-ad *allele 42 caused an indistinguishable phenotype as the losses of Tid1/Rdh54p or Ptc2p. Although the precise roles of these checkpoint adaptation genes in allowing survivor cells to recover from inactivating Cdc13p are not known, they are not simply suppressors of the ts phenotype conferred by the *cdc13-1 *allele. We could not detect any differences in the temperature dependent growth characteristics between *cdc13-1 *cells as compared to *cdc13-1 tid1*Δ or *cdc13-1 ptc2*Δ cells (Fig. S5).

Given the importance of these above genes in checkpoint adaptation, collectively our data underscore that cap-independent growth of yeast cells relies on the ability of cells to shut off checkpoint signalling. One might have suspected that once checkpoint signalling is abrogated by a mutation in one of the essential genes of the pathway (*MEC1*, *MEC3*, *RAD24*, or *RAD17*, see Fig. 3), checkpoint adaptation may not be necessary anymore and cells would readily divide and grow in Cdc13p-inactivating conditions (37°C with a *cdc13-1* allele). However, when we tested this idea by challenging *rad24* cells with a Cdc13p-loss a second time, only a small percentage of cells succeed to become dividing cultures (Fig. 5).

An efficient generation of cap-independent cells was reported to occur in cells that were engineered to lack the *RAD9 *gene as well as deletions of at least two additional genes involved in pathways that generate single-stranded DNA, namely *SGS1 *and *EXO1* 29. It is therefore tempting to speculate that checkpoint adaptation genes are required to deal with the excess single-stranded DNA that is generated after a loss of telomeric capping. This function could thus be in addition to causing the loss of phosphorylation on key checkpoint signalling proteins, but it remains unclear what this could entail. Our data therefore support the hypothesis that checkpoint adaptation might be a cancer promoting mechanism in humans and that polo-like kinases, which are required for checkpoint adaptation in yeast and human cells, could be potent anti-cancer targets 53. Further investigation of the mechanistic details of adaptation to telomeric cap loss in yeast thus has the potential to reveal new anti-cancer targets.

Finally, Cdc13p-independent cells are sensitive to the replication-interfering drug hydroxyurea (HU), yet this sensitivity is reversed upon re-introduction of wt Cdc13p (Fig. 5) and therefore is not due to mutations in checkpoint signalling genes. In parallel, upon Cdc13p-introduction, DNA damage foci are also lost in these cells (Fig. 2). In order to explain the reversible sensitivity to HU, we speculate that in cap-independent survivors, a limiting component of the DNA repair machinery is tied up at uncapped telomeres and a large amount of additional DNA damage very rapidly becomes toxic, because it is left unrepaired. Upon reintroduction of Cdc13p, capping is re-established, single stranded DNA at chromosome ends is lost and the cells regain full potential to deal with drug-induced DNA damage. This hypothesis predicts that sub-lethal interference with telomere capping in human cancerous cells would sensitize these cells to DNA damaging agents. Approaches for therapeutic uncapping are being pursued 54,55 and our results suggest that a key to their success will be to combine telomere uncapping reagents with standard DNA damage treatments.

## MATERIAL AND METHODS

### Yeast Strains and plasmids

All strains and plasmids are presented in Supplementary Tables 1 and 2, respectively. *Cdc13*Δ strains were derived from the diploid strain UCC3535 56, in which one allele of the CDC13 gene was disrupted by the natR gene, removing coding sequences + 57 to + 2361 with respect to the initiation codon of *CDC13*.

### Survivor and cap-independent cell generation

Haploid MLY100 cells containing plasmid p*cdc13-1 *were grown at 23°C for approximately 150 generations and type II survivor cells were identified by Southern-blot. Individual colonies from these type II survivors were incubated on non-selective YEPD plates at 23°C for 48 h. The colonies obtained were re-plated on YEPD every 48-72 h at gradually higher temperatures until reaching 37°C (28°C for 48 h, 30°C for 48-72 h, then 34°C for 24-72 h and finally 24 h at 37°C). The colonies obtained are passaged two times on FOA-containing plates to ensure complete loss of the plasmid p*cdc13-1. *These *cdc13*Δ - cells were then able to grow at all temperatures.

### Colony growth tests (spot dilution tests)

Strains of interest were grown in liquid media at various temperatures (as indicated in figures) until reaching exponential growth phase (OD_660nm_= 0.7 - 1.0). Cultures were then serially diluted in order to provide from 10 (minimum) to 100 000 (maximum) cells per volume plated (10 µl). After mixing and separating cells by vortexing, 10 µl aliquots of a complete dilution series (1/10 dilution factor between spots) were spotted on petri dishes containing various selective media. The resulting plates were incubated for 72 to 150 h at appropriate constant temperature until single colonies were clearly visible for positive control strains.

### Colony growth analysis 

5 ml liquid cultures of strains of interest were grown at 23°C with continuous agitation in appropriate media (e.g. YEPD plate is used for the *cdc13*Δ strains). After the cultures reached OD_660nm _= 0.5, 100 µl of the culture were spread on a marked area of a pre-heated (30°C) YEPD plates. Individual round unbudded cells were arrayed on YEPD plates as quickly as possible (max. time was 15 min) using a micromanipulator stage on a microscope. 16 cells were arrayed on one plate in a typical experiment, and a total of 64 cells of the same strain were arrayed. The plates with arrayed cells were then incubated at 30°C. Morphology and growth of cells were inspected microscopically every 2 h and colony growth progression was evaluated by counting cells as follows:

- Round unbudded cells were scored as single cells, still in G1.

- Mother cells with a bud or cells showing a dumbbell morphology were scored as 2 cells.

- Round unbudded cells attached to a mother cell with a bud were scored as 3 cells.

The plates were further incubated at 30°C up to 24 h and cells were counted again at this point. This incubation at 30°C was followed by incubation at 37°C for another 24 h. The number of cells was again registered at this point. The plates were left at 37°C for another 72 h in order to evaluate whether complete colonies could form and photos were taken at this point.

### Quantification of the rate of cdc13-independent cell generation (fluctuation test)

Yeast strains were pre-grown in 5 ml synthetic medium lacking uracil at 23°C until saturation. 2 x 10^6 ^cells were inoculated in 5 ml YEPD liquid medium and incubated at 30°C in a rotary drum with constant rotation for exactly 24 h. 10^4 ^cells were plated on YEPD plates and one plate was incubated at 23°C (for viability control) and another at 37°C (number of cap-independent cells control) for at least 72 h. The number of well-developed colonies was counted on each plate. This procedure was performed 20 times for each strain using independent colonies for their initial culture inoculation. To calculate the adaptation rate to telomere deprotection for different strains, we used the equation proposed by Luria and Delbrück 57:

**A= [-ln(N_0_/N)]/M**

**A**: Adaptation rate (number of adaptation events per cell division)

**N_0_**: Number of experiments that resulted in 0 adapted cells (number of plates with O colonies after incubation at 37°C)

**N**: Number of experiments (number of plates) 

**M**: Number of cells entering each experiment (number of cells plated per plate) 

Colonies from these type II survivors were incubated in liquid YEPD media at 23°C and 30°C until dense cultures were achieved (approximately 10 generations of growth) and then assayed for frequencies of generating Cdc13-independent cells by plating onto YEPD at 37°C and FOA plates. A total viable cell count was derived from the same cultures plated on YEPD and grown at 23°C.

### Drug sensitivity assays

For growth sensitivity assays on plates, exponentially growing cultures were 10-fold serially diluted and spotted onto YEPD or synthetic complete plates containing 0.01% MMS or 50 mM hydroxyurea. For acute exposure to MMS, exponentially growing cells were mock treated or treated with MMS by adding 0.01% MMS to the corresponding cultures for 90 min. Strains used for western blotting of Rad53p phosphorylation were treated or not with 5 µg/mL of phleomycin (phleo) of bleomycin.

### SDS PAGE and Western blot analysis

Protein extracts were prepared using a modified trichloroacetic acid method and proteins were separated by 8% SDS-PAGE as described in 28. Western blotting was performed using an in house polyclonal anti-Rad53p antibody (kindly provided by D. Durocher, Samuel Lunenfeld Research Institute, Toronto, Canada) or with a commercial polyclonal anti-Rad53p antibody (Abcam, N° ab 104232, with using a 1:500 dilution) and signals were revealed using horseradish peroxidase-conjugated anti-rabbit antibodies with the enhanced chemiluminescence (ECL) detection kit (GE-Healthcare).

### Terminal transferase and PCR telomere amplification

Purified genomic DNA was treated with terminal transferase to add a poly-C tail to chromosome ends as described 58. 100 ng of genomic DNA were heat denatured and tailed in 10 µl of 20 mM Tris-HCl pH 7.8, 50 mM KAc, 10 mM MgAc2, 1 mM dCTP with 1 U of terminal deoxynucleotidyl transferase for 30 min at 37°C. Following tailing, the enzyme was inactivated by incubating the samples for 10 min at 65°C and 5 min at 94°C. The tailing reaction was performed in 0.2 ml PCR-tubes using a thermal cycler. By using a poly-G primer and a primer hybridizing in the subtelomeric Y’ region terminal PCR products were obtained and sequenced.

## SUPPLEMENTAL MATERIAL

Click here for supplemental data file.

All supplemental data for this article are also available online at http://microbialcell.com/researcharticles/dna-damage-checkpoint-adaptation-genes-are-required-for-division-of-cells-harbouring-eroded-telomeres/.
