# Supplementary material for: DNA damage checkpoint adaptation genes are required for division of cells harbouring eroded telomeres
Source: Microb Cell. 2015 Sep 21;2(10):394–405. doi: 10.15698/mic2015.10.229 (PMC5354583; doi:10.15698/mic2015.10.229)
Supplement: Supplementary file 1 [file mic-02-394-s01.pdf]

Supplementary Material

DNA damage checkpoint adaptation genes are required for division of cells harbouring eroded telomeres.

Sofiane Y. Mersaoui, Serge Gravel, Victor Karpov, and Raymund J. Wellinger

Supplementary Method :

DSB resection activities are functional in cdc13∆ mutants:

A single colony of WT or *cdc13∆* (SGY402) cells was grown overnight for pre-culture on selective medium supplemented with Raffinose. When the culture reached to a density of $1x10^{+7}$ cells/ml (T= 0), galactose was added (final concentration of 2%) which induced the expression of the HO endonuclease and caused the introduction of an irreparable DSB cells. Cells were held with continuous shaking at 23°C and samples were collected at desired time points in 1h intervals. Genomic DNA was prepared and digested with BglII and HindIII restriction enzymes. That DNA was then analyzed by denaturing slot blot analysis with probes complementary to the resected or unresected DNA strands adjacent to the HO site of the *MAT* locus as described in [1]. Hybridization signals were quantified by ImageJ.

Legends for Supplementary Figures

Figure S1: **Generation of cdc13-independent (*cdc13Δ*) cells.** (A) Strategy for generating cap-independent *cdc13Δ* cells. After the loss of p*TLC1*, haploid cells containing plasmid p*cdc13-1* were grown for approximately 150 generations at 23°C and type II survivor cells were identified by Southern-blotting (see panel B). Cells from independent colonies were then plated on YEPD at 28˚C, 30˚C, 34˚C and finally at 37˚C. Surviving colonies were passaged two times on FOA to remove the plasmid p*cdc13-1.* These *cdc13Δ*-cells were then able to grow at all temperatures (data not shown). (B) XhoI digested DNA derived from three independent clones of survivors type I, type II as well as from final *cdc13Δ* cells generated from CPY030 was subjected to Southern blotting using a telomeric repeat DNA probe for telomeric restriction fragment analyses. M: molecular weight markers; Lanes 1, 11

wt strains; lanes 2-4: type I survivors; lanes 5-7: type II survivors; lanes 8-10: *cdc13Δ* cells. The position of normal TRFs in wt cells is indicated by an arrowhead.

Figure S2: **Mec1-dependent Chk1p phosphorylation is defective in *cdc13*Δ cells.** Exponentially growing Wild-type (WT), type II survivors (MLY113) (S) or *cdc13*Δ cells expressing a Chk1-HA fusion protein (SGY434), were left untreated or were treated with 5µg/mL phleomycin for 2h. Total protein extracts were analyzed by western blotting using an anti-HA antibody as indicated.

Figure S3: **DSB resection and repair are not abolished in *cdc13*Δ cells.** (A) An irreparable DSB was introduced in wild-type (WT) or *cdc13*Δ (Δ13) cells (SGY402 derived from RWY70D) by the expression of the HO endonuclease at T= 0. Cell samples were collected at 1h intervals, genomic DNA was prepared and analyzed by denaturing slot blot analysis with probes complementary to the resected or un-resected DNA strands adjacent to the HO site. (B) Densitometric analysis of the data presented in A. (C) The HO endonuclease was expressed in wild-type (WT) or *cdc13*Δ (Δ13) cells at T= 0. One hour later, the expression of HO was repressed to allow the repair of the DSB by HR-dependent mating-type switching process. Samples were collected at 1h intervals, genomic DNA was prepared and analyzed by Southern blot analysis. Restriction fragments corresponding to the uncut, HO-endonuclease cut and product are indicated. The band marked with an asterisk corresponds to the distal fragment and serves as a loading control.

Figure S4: **Wild type and survivor cells are not affected by overexpression of the Mec1-branch checkpoint genes.** Panels show spot dilution growth experiments of telomerase

positive (WT) or survivor type II (SII) cells of strain derived from MLY100. The indicated strains were transformed with five constructs: pGal-*MEC1*, pGal-*MEC3*, pGal-*RAD17*, pGal-*RAD24* or empty vector (vec) as a control. Cells were spotted onto YEP+ Glucose for growth control (left panels, GLUCOSE) and on YEP+ Galactose (right panels, GALACTOSE) to induce the expression of the indicated genes. Over expression of the indicated genes does not affect cell growth of telomerase positive cells or survivor cells.

Figure S5: ***Loss of PTC2* or *TID1* does not suppress the temperature sensitivity of cells harbouring a *cdc13-1 allele*:** Panels show spot dilution growth experiments of WT controls (MSY366), *tid1Δ (*MSY367), *ptc2Δ* (MSY368), *cdc13-1* (MSY369), *tid1Δ cdc13-1* (MSY370) and *ptc2Δ cdc13-1* (MSY371) cells, all derived from MLY100. The indicated strains were spotted onto YEPD for growth control at 23°C (left panels) and on YEPD at 28°C and 30°C (middle and right panels). Disruption of the indicated genes does not suppress cell growth defect of conferred by the *cdc13-1* mutation at restrictive temperature.

Supplementary Table 1: Strains used in this study

Strain	Genotype	Source
UCC3535	*Mat A/alpha, tlc1Δ::LEU2/TLC1 ade2-101/ade2-101 leu2-Δ1/ leu2-Δ1 lys2-801/lys2-801 ura3- 52/ura3-52 trp1-Δ63/ trp1-Δ63 his3Δ200/ his3Δ200 DIA5-1/DIA5-1*	[2]
MLY100*	*Mata/alpha, TLC1/tlc1Δ::LEU2,* ***CDC13/cdc13Δ::NatR****,* ***VR-ADE2-T/VR-ADE2-T****, ura3-52/ura3-52, lys2-801/lys2-801, ade2-101/ade2-101, trp1-Δ63/trp1-Δ63, his3-Δ200/his3-Δ200, leu2-Δ1/leu2-Δ1* (from UCC3535).	[3]
MLY112	***Survivor Type II****, Mata tlc1Δ::LEU2 cdc13Δ::NatR VR-ADE2-T ura3-52 lys2-801 ade2-101 trp1-Δ63 his3-Δ200 leu2-Δ1,* **+ p*cdc13-1***.	This study
MLY113	***Survivor Type II***, Isogenic to **MLY112.**	This study
MLY122	***Cdc13-independent*** *, Mata tlc1Δ::LEU2 cdc13Δ::NatR VR-ADE2-T ura3-52 lys2-801 ade2-101 trp1-Δ63 his3-Δ200 leu2-Δ1.* **Derived from MLY112**	This study
MLY123	***Cdc13-independent***. Mat A tlc1::LEU2 cdc13::NatR VR-ADE2-T ura3-52 lys2-801 ade2-101 trp1-del63 his3del200 leu2-del1 Isogenic to MLY122	[3]
VKY12	***Survivor Type II****, Mata, tlc1Δ::LEU2, cdc13Δ::NatR,* ***ptc2Δ::KanMX4****, VR-ADE2-T, ura3-52, lys2-801, ade2-101, trp1-Δ63, his3-Δ200, leu2-Δ1,* **+ p*cdc13-1***	This study
VKY19	***Survivor Type II****, Mata, tlc1Δ::LEU2, cdc13Δ::NatR,* ***tid1Δ::KanMX4****, VR-ADE2-T, ura3-52, lys2-801, ade2-101, trp1-Δ63, his3-Δ200, leu2-Δ1, +* p*cdc13-1*	This study
VKY20	*Mata, cdc13Δ::NatR, VR-ADE2-T, ura3-52, lys2-801, ade2-101, trp1-Δ63, his3-Δ200, leu2-Δ1,* **+ p*cdc13-1.***	This study
CPY030	*Matalpha tlc1Δ::KnMX cdc13Δ::NatR bar1Δ::LEU2 ura3-52, lys2-801, ade2-101, trp1-Δ63, leu2-Δ1****+ pTLC1TRP + pcdc13-1***<br>***Cdc13-independent cells****, Matalpha tlc1Δ::KnMX cdc13Δ::NatR*	This study
MSY054	*bar1Δ::LEU2, ura3-52, lys2-801, ade2-101, trp1-Δ63, leu2-Δ1* ***rad24***$^{-}$ (derivative of CPY030)	This study
RWY801	*Mata tlc1Δ::KnMX cdc13Δ::NatR bar1Δ::LEU2 trp1-1 ura3-1 leu2-3,112 his3-11,15 can1-100* ***RFA1-YFP***	This study

RWY70D	*MatA-inc/Matalpha hmlΔ::ADE1/hmlΔ::ADE1 hmrΔ::ADE1/hmrΔ::ADE1 HOΔ/HOΔ ade1-100/ade1-100 leu2-3,112/leu2-3,112 trp1Δ::hisG/trp1Δ::hisG lys5/lys5 ura3-52/ura3-52 ade3::GAL::HO/ade3::GAL::HO cdc13Δ::NatR/CDC13 tlc1Δ::LEU2/TLC1*	This study
SGY402**	*MATalpha hmlΔ::ADE1 hmrΔ::ADE1 HOΔ ade1-100 leu2-3,112 trp1Δ::hisG lys5 ura3-52 ade3::GAL::HO cdc13Δ::NatR tlc1Δ::LEU2* **+ p*cdc13-1* + p*TLC1***	This study
CPY821	*Mata tlc1Δ::KnMX cdc13Δ::NatR bar1Δ::LEU2 trp1-1 ura3-1 leu2-3,112 his3-11,15 can1-100* ***RAD52-YFP***	This study
SGY446	*Mata tlc1Δ::LEU2 cdc13Δ::NatR* ***tel1Δ::kanMX*** *VR-ADE2-T ura3-52 lys2-801 ade2-101 trp1-del63 his3del200 leu2-del1* **(derivative of MLY112)**	This study
SGY434	*Mata tlc1Δ::LEU2 cdc13Δ::NatR VR-ADE2-T ura3-52 lys2-801 ade2-101 trp1-del63 his3del200 leu2-del1* ***CHK1-3xHA::HIS3MX, (*****derivative of MLY112*)*	This study
MSY366	***WT,*** *VR-ADE2-T*, *ura3-52, lys2-801, ade2-101, trp1-Δ63, his3-Δ200, leu2-Δ1*	This study
MSY367	***tid1Δ::KanMX4***, *VR-ADE2-T, ura3-52, lys2-801, ade2-101, trp1-Δ63, his3-Δ200, leu2-Δ1*	This study
MSY368	***ptc2Δ::KanMX4***, *VR-ADE2-T, ura3-52, lys2-801, ade2-101, trp1-Δ63, his3-Δ200, leu2-Δ1*	This study
MSY369	*cdc13Δ::NatR, VR-ADE2-T, ura3-52, lys2-801, ade2-101, trp1-Δ63, his3-Δ200, leu2-Δ1,* **+ p*cdc13-1***	This study
MSY370	*cdc13Δ::NatR,* ***tid1Δ::KanMX4,*** *VR-ADE2-T, ura3-52, lys2-801, ade2-101, trp1-Δ63, his3-Δ200, leu2-Δ1,* **+ p*cdc13-1***	This study
MSY371	*cdc13Δ::NatR,* ***ptc2Δ::KanMX4,*** *VR-ADE2-T, ura3-52, lys2-801, ade2-101, trp1-Δ63, his3-Δ200, leu2-Δ1,* **+ p*cdc13-1***	This study
MSY421	***Survivor Type II***, *Mata, tlc1Δ::LEU2, cdc13Δ::NatR,* ***cdc5-ad:HIS3***, *VR-ADE2-T, ura3-52, lys2-801, ade2-101, trp1-Δ63, his3-Δ200, leu2-Δ1,* **+ p*cdc13-1***	This study

* For the process of generating *cdc13Δ* strains, the diploid strain MLY100 was transformed with plasmid pcdc13-1 and *cdc13Δ::natR* haploid spores containing p*cdc13-1* were isolated (ex CPY030).

** SGY402 was considered WT and then was processed to lose p*TLC1* and p*cdc13-1* to generate cdc13Δ cells for Fig. S3 as described in the Methods.

Supplementary Table 2: Plasmids used in this study

Name	Description	Source
p*cdc13-1*	CEN, *URA3*, *cdc13-1 (ts)*	[3]
p*TLC1*	pRS314, CEN, *TRP1*, *TLC1*	[4]
pRS400	Containing the KanMX4 sequence for deletion	[5]
pGal-Empty	pYES2 (Vector containing a galactose inducible promoter)	Stratagene Inc.
pGal-MEC3	From pGal-Empty	This study
pGal-MEC1	From pGal-Empty	This study
pGal-RAD17	From pGal-Empty	This study
pGal-RAD24	From pGal-Empty	This study

<u>Supplementary References:</u>

1. Sugawara N, Haber JE (**1992**). Characterization of double-strand break-induced recombination: homology requirements and single-stranded DNA formation. **Mol. Cell. Biol.** 12(2): 563-575. PMC364230

1. Sugawara N, Haber JE (**1992**). Characterization of double-strand break-induced recombination: homology requirements and single-stranded DNA formation. **Mol Cell Biol** 12(2): 563-575. doi.

2. Wellinger RJ, Ethier K, Labrecque P, Zakian VA (**1996**). Evidence for a new step in telomere maintenance. **Cell** 85(3): 423-433. doi: http://dx.doi.org/10.1016/S0092-8674(00)81120-4.

3. Larrivee M, Wellinger RJ (**2006**). Telomerase- and capping-independent yeast survivors with alternate telomere states. **Nat Cell Biol** 8(7): 741-747. doi: 10.1038/ncb1429.

4. Bah A, Bachand F, Clair E, Autexier C, Wellinger RJ (**2004**). Humanized telomeres and an attempt to express a functional human telomerase in yeast. **Nucleic Acids Res** 32(6): 1917-1927. doi: 10.1093/nar/gkh511.

5. Brachmann CB, Davies A, Cost GJ, Caputo E, Li J, Hieter P, Boeke JD (**1998**). Designer deletion strains derived from Saccharomyces cerevisiae S288C: a useful set of strains and plasmids for PCR-mediated gene disruption and other applications. **Yeast** 14(2): 115-132. doi: 10.1002/(SICI)1097-0061(19980130)14:2<115::AID-YEA204>3.0.CO;2-2.

Figure S1

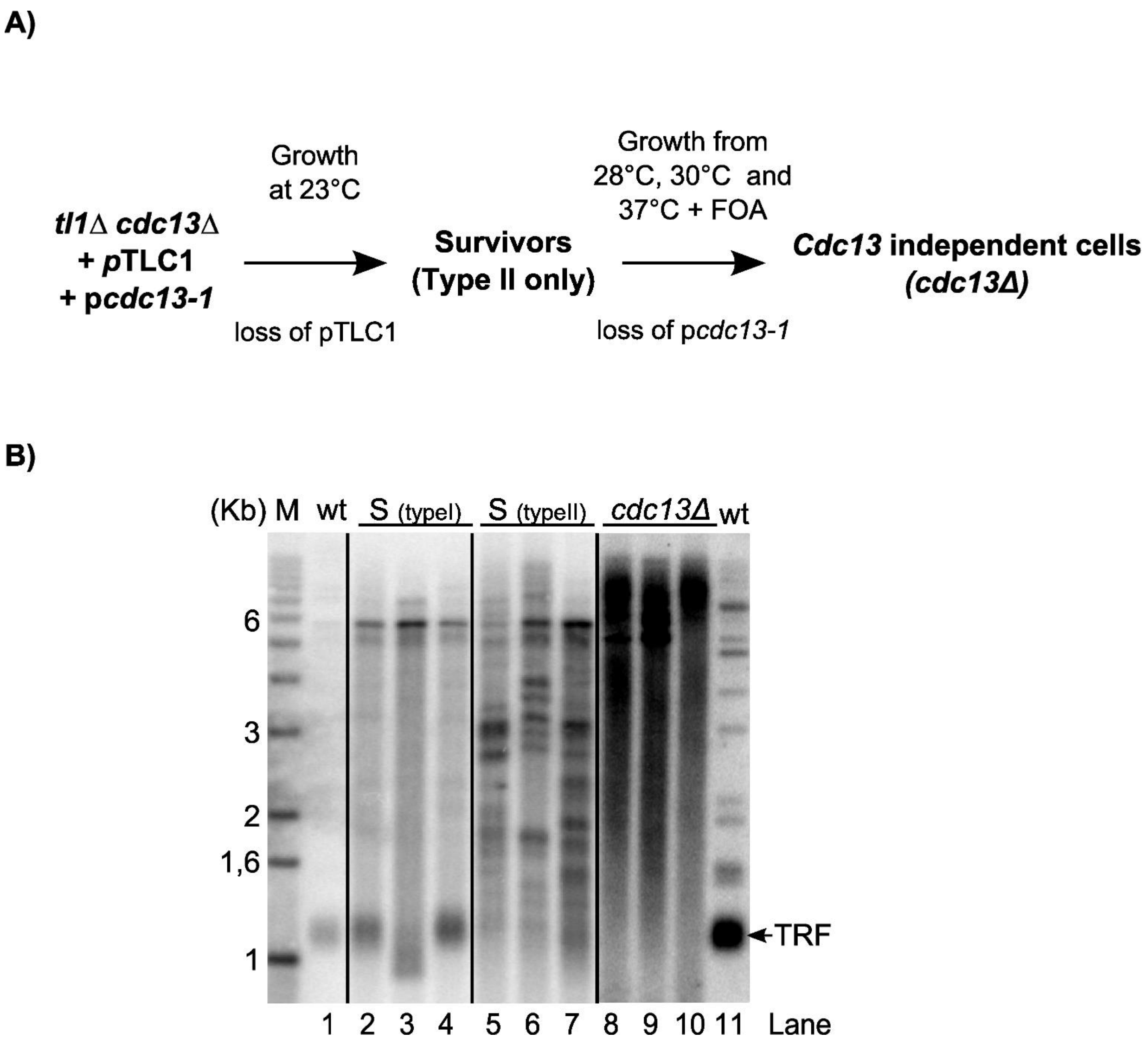
A)
tl1Δ cdc13Δ
+ pTLC1
+ pcdc13-1
Growth
at 23°C
loss of pTLC1
Survivors
(Type II only)
Growth from
28°C, 30°C and
37°C + FOA
loss of pcdc13-1
Cdc13 independent cells
(cdc13Δ)
B)
(Kb) M
wt
S (typeI)
S (typeII)
cdc13Δ
wt
6
3
2
1,6
1
TRF
1 2 3 4 5 6 7 8 9 10 11 Lane

Figure S2

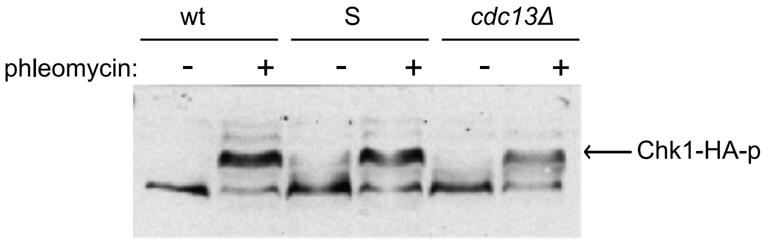

Figure S3

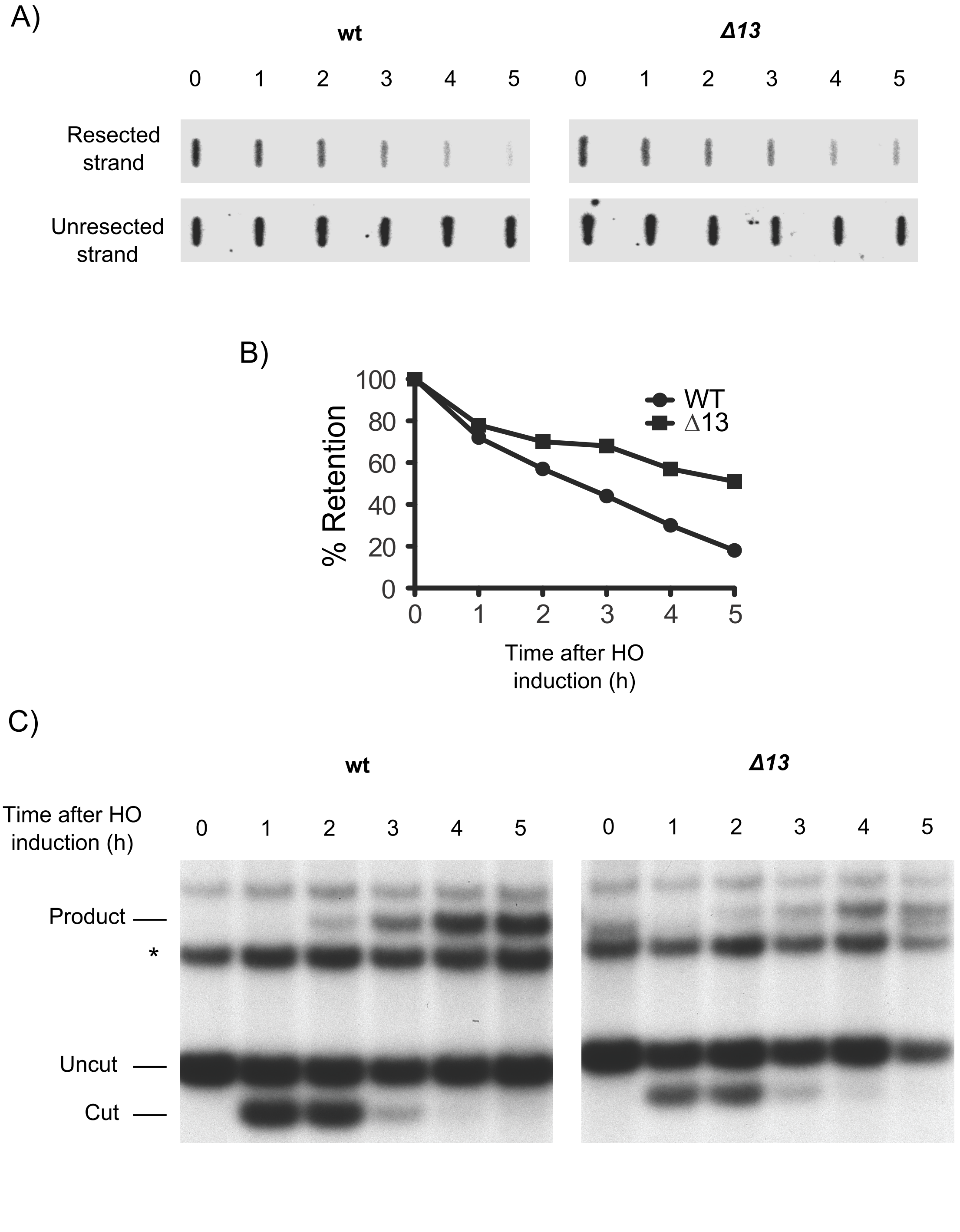
A)
wt
Δ13
0 1 2 3 4 5 0 1 2 3 4 5
Resected strand
Unresected strand
B)
% Retention
100
80
60
40
20
0
WT
Δ13
0 1 2 3 4 5
Time after HO induction (h)
C)
wt
Δ13
Time after HO induction (h)
0 1 2 3 4 5 0 1 2 3 4 5
Product
*
Uncut
Cut

Figure S4

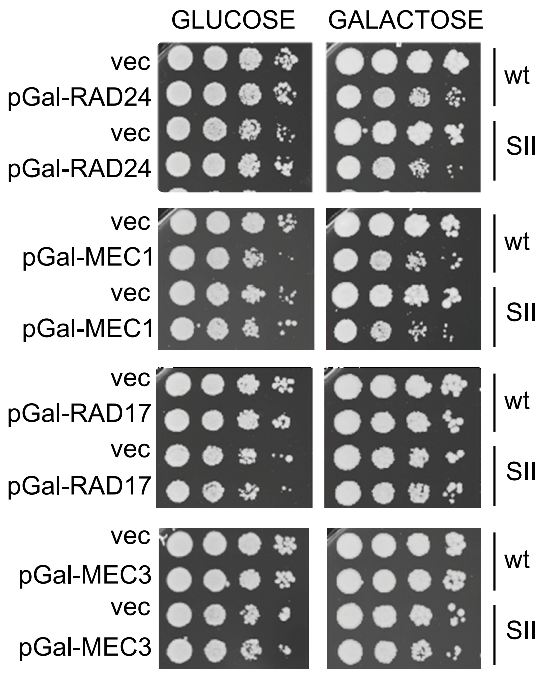
GLUCOSE
GALACTOSE
vec
pGal-RAD24
vec
pGal-RAD24
wt
SII
vec
pGal-MEC1
vec
pGal-MEC1
wt
SII
vec
pGal-RAD17
vec
pGal-RAD17
wt
SII
vec
pGal-MEC3
vec
pGal-MEC3
wt
SII

Figure S5:

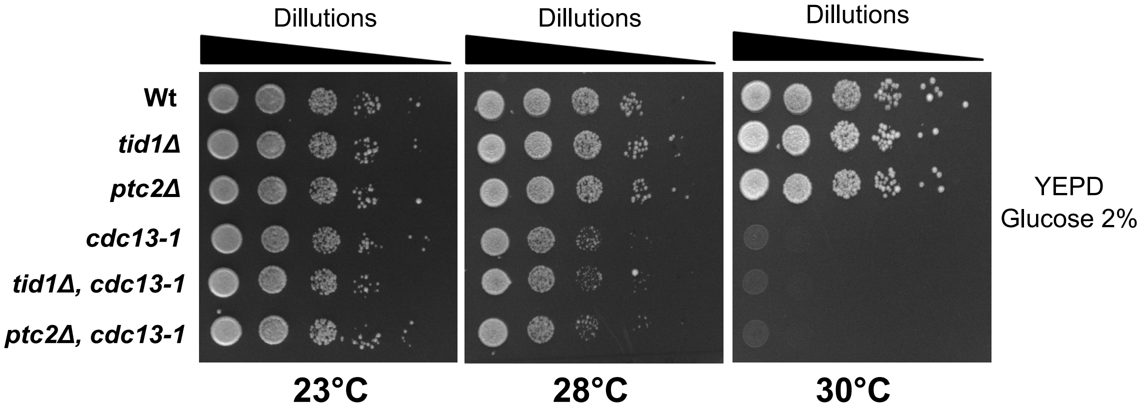
Dillutions
Dillutions
Dillutions
Wt
tid1Δ
ptc2Δ
cdc13-1
tid1Δ, cdc13-1
ptc2Δ, cdc13-1
YEPD
Glucose 2%
23°C
28°C
30°C
